# New frontiers on the molecular underpinnings of hypospadias according to severity

**DOI:** 10.1080/2090598X.2020.1760589

**Published:** 2020-05-24

**Authors:** Coriness Piñeyro-Ruiz, Horacio Serrano, Marcos R. Pérez-Brayfield, Juan Carlos Jorge

**Affiliations:** a Department of Anatomy and Neurobiology, School of Medicine, University of Puerto Rico, San Juan, Puerto Rico, USA; b Department of Internal Medicine and Department of Biochemistry, University of Puerto Rico, San Juan, Puerto Rico, USA; c Department of Surgery, Section of Urology, School of Medicine, University of Puerto Rico, San Juan, Puerto Rico, USA

**Keywords:** Hypospadias, aetiology, urogenital, molecular biology, human genetics

## Abstract

Hypospadias, which is characterised by the displacement of the urethral meatus from its typical anatomical location in males, shows various degrees of severity. In this systematic review, we surveyed our current understanding of the genetics of isolated hypospadias in humans according to the severity of the condition. We found that sequencing and genotyping approaches were the preferred methods of study and that single nucleotide polymorphisms were the most common finding associated with hypospadias. Most genes fell into four gene-pathway categories related to androgens, oestrogens, growth factors, or transcription factors. Few hypospadias studies classify their findings by severity. Taken together, we argue that it is advantageous to take into consideration the severity of the condition in search of novel candidates in the aetiology of hypospadias.

**Abbreviations:** AR: androgen receptor; ATF3: activating transcription factor 3; BMP4: bone morphogenetic protein 4; BMP7: bone morphogenetic protein 7; CYP17: steroid 17-alpha-hydroxylase/17,20 lyase; CYP1A1: cytochrome P450 1A1; CYP3A4: cytochrome P450 3A4; CNVs: copy number variants; DGKK: diacylglycerol kinase kappa; ESR1: oestrogen receptor 1; ESR2: oestrogen receptor 2; FGF8: fibroblast growth factor 8; FGF10: fibroblast growth factor 10; FGFR2: fibroblast growth factor receptor 2; HOXA4: homeobox protein Hox-A4; HOXB6: homeobox protein Hox-B6; HSD17B3: hydroxysteroid 17-beta dehydrogenase 3; MAMLD1: mastermind-like domain-containing protein 1; SF-1: splicing factor 1; SHH: sonic hedgehog; SNPs: single nucleotide polymorphisms; SOX9: SRY-box 9; SRD5A2: steroid 5 alpha-reductase 2; SRY: sex-determining region Y protein; STAR: steroidogenic acute regulatory protein; STARD3: StAR-related lipid transfer protein 3; STS: steryl-sulfatase; WT1: Wilms tumour protein; ZEB1: zinc finger oestrogen-box binding homeobox 1

## Introduction

There are several classification systems describing the position of the opening of the urethral meatus in hypospadias according to the severity of the condition. While classification as mild or severe hypospadias may guide the surgeon to opt for a given surgical technique and approach [1], from a research standpoint, the specific anatomical location of the urethral meatus may provide essential insights about the timeframes and mechanisms of actions that were altered during embryonic and/or fetal development of the urethral plate. This systematic review identifies the state of knowledge from genetics and molecular biology studies on the aetiology of hypospadias and points at plausible clinical applications that could predict expected surgical outcomes when additional genetic information according to the severity of the condition becomes available.

The urethral meatus in males born with hypospadias can be located anywhere in the ventral plane of the penis, the scrotum, or the raphe line in the perineum. Hypospadias can also be accompanied by a ventral chordee or penile curvature and incomplete formation of the foreskin. The penile skin defect is characterised by excess skin in the distal dorsal part of the penis, often referred to as a dorsal hood, and a ventral deficiency [2–6]. Because of the complexities of hypospadias severities, its aetiology is considered to be multifactorial [3,7–11].

Classification of hypospadias severity follows the anatomical location of the urethral meatus. Clinton K. Smith [12] was the first to classify hypospadias according to the location of the urethral meatus. He classified hypospadias as first degree (opening is situated in the distal one-third of the penis); second degree (proximal two-thirds of the penis to the penoscrotal junction); and third degree (point backward to the perineum). Thereafter, Schaefer and Erbes [5] employed Smith’s degrees as glandular (first degree), penile (second degree), and perineal (third degree). Although these classification systems based on anatomy are useful, clinicians noted that surgical correction of ventral foreskin tethering and/or significant chordee might displace the urethral meatus to a different location, which may lead to misclassification of severity. Therefore, the Sheldon and Duckett [6] classification system, modified after Barcat’s classification, considers the meatus position after chordee has been released. They classified hypospadias as anterior hypospadias, described as glandular (located near the tip of the glans), subcoronal (located just below the coronal sulcus; middle hypospadias (distal penile and midshaft); and posterior hypospadias (proximal penile and penoscrotal meatus), scrotal (located in the scrotum), and perineal (located below the scrotum and perineum). Hypospadias can also be grouped as mild (Type I), moderate (Type II), and severe (Type III) hypospadias, which can be grouped further as mild (Type I) and severe (Type II and III) hypospadias. About 70% of cases are mild, and the remaining 30% are severe [8–10,13,14]. Given the tremendous advances in molecular biology techniques over the last few decades, the present review aimed to highlight the emerging scenarios with regard to key biological factors that have been related to hypospadias according to the severity of the condition.

## Methods

The literature search consisted of published scientific literature for human studies that involved the study of isolated hypospadias by employing molecular biology techniques using the PubMed database (https://www.ncbi.nlm.nih.gov/pubmed). A search was performed on 1 February 2019, yielding 340 results. PubMed query read as follows:

(hypospadias AND (gene OR protein OR metabolite OR molecular) AND English[Language] NOT syndrome NOT disorders of sex development NOT Klinefelter NOT review [Publication Type] NOT case reports [Publication Type] NOT clinical trial [Publication Type]).

**Figure 1.**
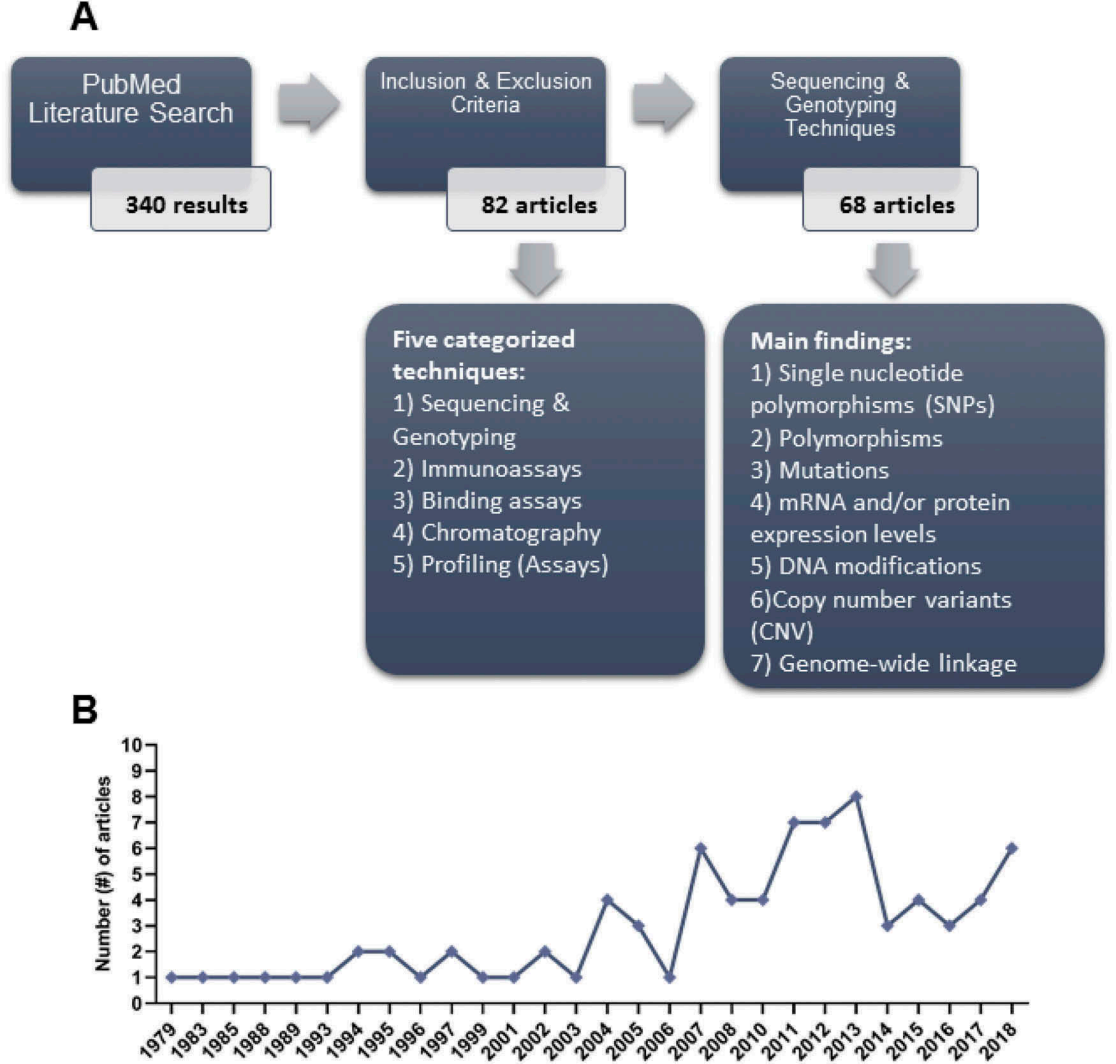
Algorithm for the systematic review. (a) A total of 340 studies evaluated isolated hypospadias in humans employing molecular biology as the main experimental approach. After inclusion and exclusion criteria, 82 studies underwent full review to assess methodological approach and from these, 68 studies took advantage of sequencing and genotyping approaches. (b) Trend line in the frequency of studies that employed molecular biology approaches in the search for hypospadias’ aetiology (1979–2018)

Restriction on the year of publication was not used. Search results were examined through titles and abstracts. The following exclusion criteria were implemented: (1) study with hypospadias patients with other conditions and/or syndromes; (2) study evaluated animals only; (3) studies with female patients; and (4) meta-analysis studies. After the identification and exclusion of articles, 82 articles remained. Figure 1(a) depicts the literature search algorithm for this systematic review, while Figure 1(b) shows a plateau in the frequency of molecular biology studies in search of hypospadias’ aetiology that began in the year 2007.

## Findings

**Figure 2.**
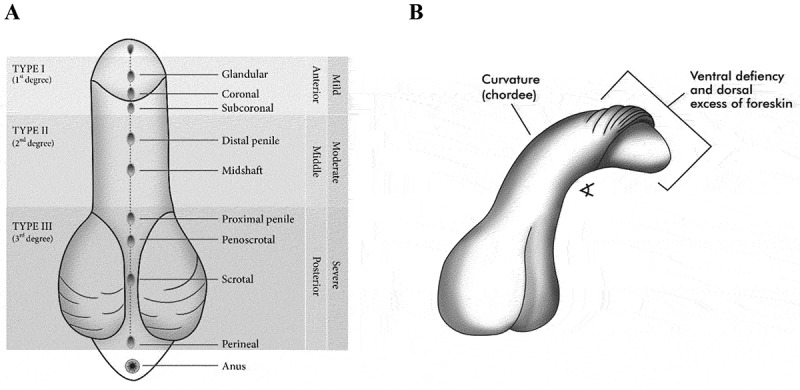
Anatomical features of hypospadias. (A) The anatomical position of the urethral meatus in males is used as the criterion to classify the severity of hypospadias. Several classification systems of hypospadias' severities are depicted. Illustration modified after Piñeyro-Ruiz C, Chorna NE, Pérez-Brayfield MR, Jorge JC. Severity-Dependent Profile of the metabolome in hypospadias. Front Pediatr. 2020 [Epub ahead of print]. 9 pages. http://doi.org/10.3389/fped.2020.00202. Copyright © 2020 Piñeyro-Ruiz, Chorna, Pérez-Brayfield and Jorge. [This is an open access article distributed under the terms of the Creative Commons Attribution License (CC BY). The use, distribution or reproduction in other forums is permitted, provided the original author(s) and the copyright owner(s) are credited and that the original publication in this journal is cited, in accordance with accepted academic practice. No use, distribution or reproduction is permitted which does not comply with these terms.] Modifications from original: grey tones and elimination of sample size numbers for some anatomical locations of the urethral meatus. (B) Hypospadias can be accompanied by penile curvature of varying degrees, ventral deficiency, and dorsal excess of foreskin

We assessed the study of hypospadias’ aetiology through molecular biology techniques according to the reported location of the urethral meatus. Figure 2(a) shows various classification systems to account for the severity of the condition that describes the anatomical location of the urethral meatus, albeit degree of penile curvature and appearance of the foreskin can also be affected in this congenital condition (Figure 2(b)).

**Figure 3.**
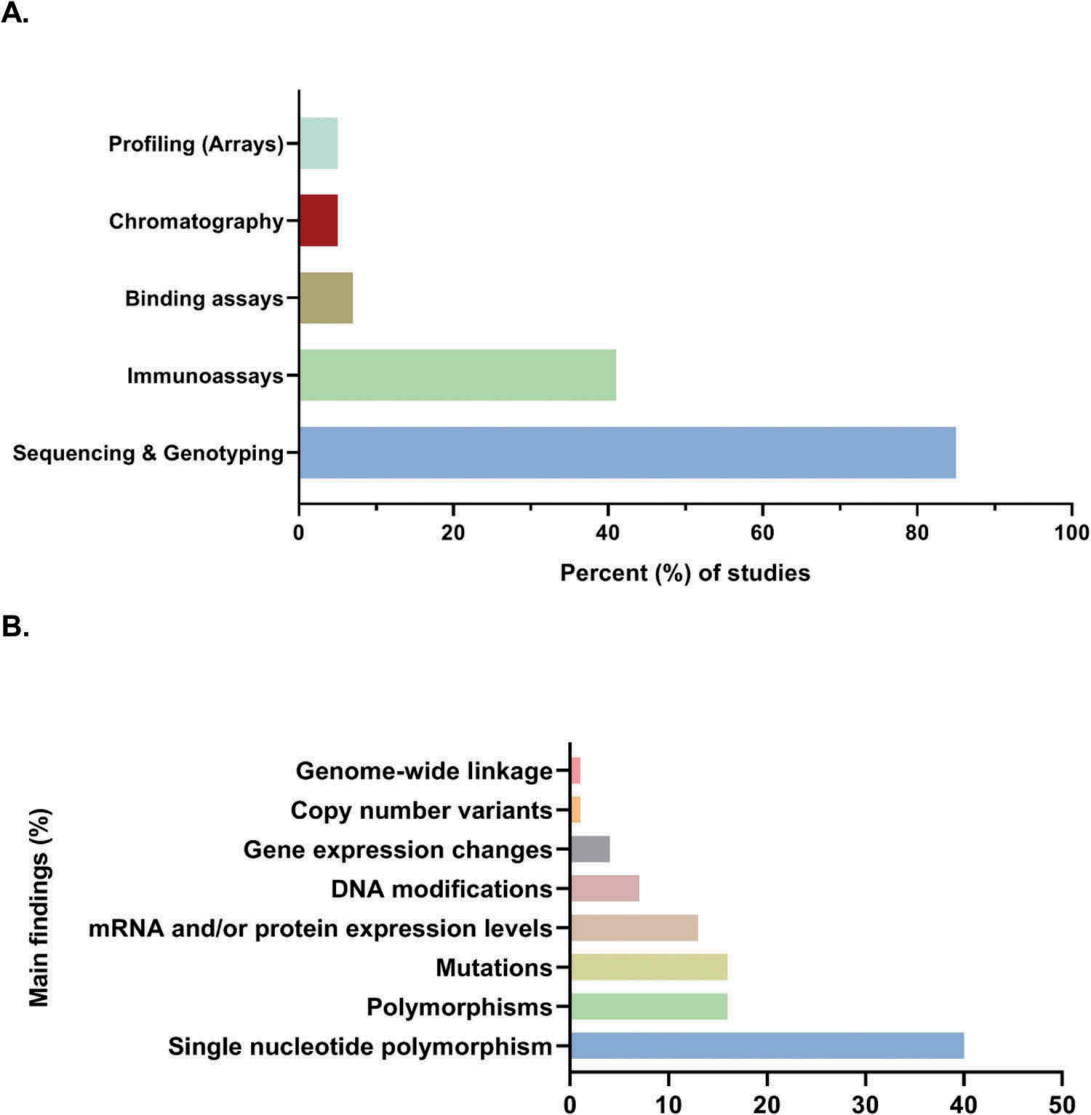
Genetics and molecular biology of hypospadias. (a) Sequencing and genotyping have been the most common methodological approach and (b) single nucleotide polymorphisms (SNPs) have been the most commonly reported finding in the search of hypospadias’ aetiology (*n* = 68 studies)

Figure 3(a) shows the percentage distribution of studies *per* experimental molecular approach: sequencing and genotyping (*n*= 70), immunoassays (*n*= 34), binding assays (*n*= 6), chromatography (*n*= 4), and profiling arrays (*n*= 4). Sequencing and genotyping were the most common experimental approaches that have been employed in search of hypospadias’ aetiology according to severity (*n*= 68). Figure 3(b) shows the percentage distribution of these studies according to the reported main finding: single nucleotide polymorphisms (SNPs; *n*= 27), polymorphisms (*n*= 11), mutations (*n*= 11), changes in mRNA and/or protein expression levels (*n*= 9), DNA modifications (*n*= 5), changes in gene expression (*n*= 3), copy number variants (CNVs; *n*= 1), and genome-wide linkage (*n*= 1) for a total of 68 studies.

We found that most genes fell into four gene-pathway categories related to androgens, oestrogens, growth factors, or transcription factors. An association with hypospadias for these genes was attributed to all hypospadias severities, amongst each severity or did not specify severity. Genes related to androgen pathways have traditionally received attention in the search of hypospadias aetiology since, clearly, the development of the male external genitalia depends on androgen signalling. One of the most studied genes has been the androgen receptor (AR) gene. Nevertheless, confounding effects have been reported. SNPs located in the promoter region of this gene have recently been identified in all hypospadias’ severities [15], and some studies report an association with severe hypospadias [16,17]. A significant increase of CAG repeats has either been found in all hypospadias severities [18,19], or none at all [20,21]. GGN repeats in patients with moderate hypospadias have also been reported [20]. Genes that are oestrogen-responsive have also been associated with hypospadias. The cyclic AMP-dependent transcription factor (*ATF3*) gene is one example. An upregulation in gene expression of this gene and specific SNPs have been found in all types of hypospadias [22]. On the other hand, specific polymorphisms have been detected for mild and severe hypospadias [23], but not for moderate hypospadias. A missense mutation in exon 3 has been identified for moderate hypospadias but not for mild and severe hypospadias [24]. For genes associated with growth factors during development, associations have been found concerning all hypospadias severities. Mutations have been identified for bone morphogenetic protein 4 and 7 (*BMP4/7*), and homeobox protein *Hox-A4* and *Hox-B6* [25]. SNPs have been associated with *BMP4, HOXA4*, sex-determining region Y protein (SRY)-box 9 (*SOX9*), fibroblast growth factor 8 and 10 (*FGF8* and *FGF10*), and for fibroblast growth factor receptor 2 (*FGFR2*) [25–27]. For transcription factor genes, the Wilms tumour protein (*WT1*) gene has been the most studied. *WT1* SNPs have been associated with all types of hypospadias [28], and a significantly increased risk associated with SNPs was found for severe hypospadias [29]. Mutations identified have been identified for all types of hypospadias [28], as well as for specific mutations for severe hypospadias [29]. Additional findings, according to hypospadias’ severity, are detailed in Table 1 [15–71].

**Table 1.** Four gene-pathway categories related to hypospadias according to severity

Gene	General hypospadias	Mild hypospadias	Moderate hypospadias	Severe hypospadias
Androgen pathways				
*AR*	New identified SNPs in promoter region [30]. Significantly increased mRNA and protein levels in prepuce samples [31]. Significant increase of CAG repeats [18,29] and no significant differences [20,21]. Mutations associated [17,32] and no mutations identified [21,33,34]. Silent polymorphism identified in three patients [24].	Pathogenic mutations found [35]. Increased gene methylation and decreased gene expression [27].	Significant decrease in mRNA expression in urethral mucosa [36]. Significantly high GGN repeats [20]. Missense mutation in one patient [37].	Pathogenic mutations found [24,35,41]. SNPs significantly associated with increased risk [38]. Significantly high protein expression levels in prepuce samples [28]. Single nucleotide substitution found in one patient [16].
*SRD5A2*	New identified mutations [19,26,39]. Polymorphisms significantly associated [19,28,40,66] and to reduced risk [41]. Leu–Ala haplotype increases the risk [19]. A49 T, R227Q and TA repeat polymorphisms were not found as a risk [42]. SNPs identified were associated with increased risk [17,43], when estrogen exposure occurs [44], and no association [38,45].	Pathogenic mutations found [35].		SNPs significantly associated with increased risk [46,47]. Mutations associated with the condition were identified [41,48]. Pathogenic mutations found [34,35].
*CYP17*	Mutations identified [49]. No associations for polymorphisms identified in CYP17-A1/A2 [49].			
*CYP1A1*	Polymorphism identified was associated with decreased risk [50] and not associated [51].			
*CYP3A4*			SNPs identified associated with increased risk [43].	SNPs identified associated with increased risk [43].
*MAMLD1*	SNPs identified significantly associated [52–54]. Polymorphism detected [23].			Identification of one new missense mutation [54].
*SF-1*				Mutation identified [34,55].
*SRY*	No risk association for CNVs [56]. No mutation found [17].			
*STS*	SNPs identified associated with increased risk [43].	SNPs identified associated with increased risk [46].		SNPs identified associated with increased risk [46].
*STAR*	SNPs identified associated with increased risk [43].			SNPs identified associated with increased risk [46].
Oestrogen pathways				
*ATF3*	Upregulation of gene expression [22]. SNPs identified associated with increased risk [44,45,57]. After estrogen exposure to Hs68 cells, a significant increase in protein expression and promoter activity [58]. Increased ATF3 mRNA expression levels in prepuce samples [59].	Polymorphisms detected [23]. Increased protein expression in the urethral plate and subcutaneous tissue [60].	Missense mutation in exon 3 identified [57]. Significant difference in gene expression [61].	Polymorphisms detected [23]. 3ʹ-UTR polymorphism identified [57]. Significant difference in gene expression [61].
*ESR1*	SNPs and haplotypes identified associated with increased risk [44,62] and no associations [45,63].	Decreased mRNA expression in foreskin samples [64].		Decreased mRNA expression in foreskin samples [64].
*ESR2*	SNPs identified associated with increased risk [33,36,37]. No significant haplotype association [23].	Decreased mRNA expression in foreskin samples [64].		Decreased mRNA expression in foreskin samples [64].
Growth factors pathways				
*BMP4*	Mutations identified [25]			
*BMP7*	Significant association of identified SNPs [65] and no identified SNPs [26]. Mutations identified [25].			
*FGF8*	Significant association of identified SNPs [26]			
*FGF10*	Significant association of identified SNPs [65] and no identified SNPs [26].			
*FGFR2*	Significant association of identified SNPs [26]			
*HOXA4*	Significant association of identified SNPs [15]. Mutations identified [25]			
*HOXB6*	Mutations identified [25]			
Transcription factors pathways				
*DGKK*	Significant association of identified SNPs [67–69].			
*SOX9*	Significant association of identified SNPs [17]. No mutations identified [17].			
*WT1*	Significant association of identified SNPs [17]. Mutations identified [17].	Low frequency of polymorphism identified [70]. Nucleotide transition 390 C–T in exon 1 [71].	High frequency of polymorphism [70]. Mutations identified [70]. Nucleotide transition 390 C–T in exon 1 [71].	Higher frequency of polymorphism [70]. Identified mutations [70] SNP significantly associated with increased risk [65].

## Discussion

It is of great significance that efforts to uncover the multifactorial aetiologies of hypospadias have taken advantage of ongoing state-of-the-art technological advances in genetics and molecular biology. It is promising that over the past decade, there have been growing research efforts to study hypospadias’ aetiology according to the severity of the condition [29,38,46,68]. This emerging work points at important severity-dependent correlations related to the *AR* gene, the zinc finger oestrogen-box binding homeobox 1 (*ZEB1*) gene, CAG repeats, the steroid sulfatase (*STS*) gene and the diacylglycerol kinase kappa (*DGKK*) gene, respectively. Further work is required to study plausible links between these biological factors and developmental events of the urethral plate.

It is becoming increasingly clear that genetic variants, including insertions, deletions, polymorphisms, and CNVs, are related to hypospadias. SNPs are common single-nucleotide variations [72], which can be used to track the inheritance of diseases. Some SNPs that have been related to hypospadias include genes involved in steroid-dependent pathways such as the AR, hydroxysteroid 17-beta dehydrogenase 3 (*HSD17B3*), StAR-related lipid transfer protein 3 (*STARD3*), steroid 5 alpha-reductase 2 (*SRD5A2*) [17,30,38,43–47]; oestrogen-dependent genes such as oestrogen receptor 1 and 2 (*ESR1* and *ESR2*) and *ATF3* [45,54,57,62,63,66,73]. An association and/or risk have also been reported for SNPs among genes that are critical during embryogenesis such as *BMP7, FGF8* and *FGF10*, sonic hedgehog (*SHH), SOX9*, amongst others [17,26,65]. Important to note is that up-to-date, <10% of hypospadias cases are attributed to genetic hereditability [4,8,9,74,75]. Therefore, it would be advantageous to investigate further into the downstream products of these genes.

With regard to severity-specific findings, it is worth noting that Singh et al. [46] found an association for SNP rs17268974 in the *STS* gene with Type II hypospadias and for seven SNPs in the *STS* gene and two in the *STARD3* gene with Type III hypospadias, genes involved in steroid metabolism, whereas, no association was found for Type I hypospadias for these SNPs. In addition, SNP rs5919436 in the *AR* gene was found to have a significant association with Type III hypospadias, but not for Type I and Type II hypospadias [38]. mRNA and protein expression levels of the *ZEB1* gene, a gene responsive to oestrogen that has been associated to isolated hypospadias, were found significantly upregulated in patients with severe hypospadias in comparison to patients with mild hypospadias [28]. In terms of DNA modifications, CAG repeats were found to be significantly higher in patients with severe hypospadias compared with non-severe patients [29]. Despite significant advances in our current understanding of the molecular underpinnings of hypospadias, further work is required to reduce the occurrence of this urogenital condition, especially the most severe forms [1,76,77].

### Clinical implications

This systematic review raises several observations relevant to the clinical management of hypospadias. First, although it has been long-recognised that suboptimal androgen signalling is associated with hypospadias, molecular studies are now refining such association. For instance, SNPs have been reported more frequently among severe than milder forms. Second, with regard to oestrogen signalling, available data do not seem able to discriminate between severity degrees. Third, there is a gap in knowledge about plausible associations between key growth factors and the severity of the condition. Future research in this area is warranted. Fourth, disruption of key transcription factors that differentiate the indifferent gonad into testis seems to participate across severities indicating an early disruption in development, which makes it challenging to identify in clinical studies.

Even for the experienced paediatric urologist, determining the precise location of the urethral opening may only be feasible at the time of surgery, as it is influenced by the curvature of the penis, the configuration of redundant skin, and the characteristics of the urethral lumen. As concerted efforts are made for shared decision-making in paediatric care [78], it would be advantageous to predict the risk of complications (i.e. fistulae) in order to provide full disclosure of relevant information to affected parents with regard to expected surgical outcomes before the urethroplasty procedure. In this context, information from genetic screening for specific mutations as the ones reported here added to our clinical assessment of the severity of the hypospadias may predict the most likely surgical outcomes with regard to urinary function and cosmesis. In fact, it has been shown that parental decisional regret is influenced by surgical outcomes [79]. Interestingly, the level of agreement between parents and the paediatric urologist concerning satisfaction with surgery is greater for severe rather than for mild cases [80,81]. Taken together, in the future, additional information provided by molecular genetics screening prior to the urethroplasty procedure may nurture open discussions with parents about expected outcomes.

### New promising molecular approaches

Novel high-throughput methodological approaches, the so-called ‘omics’ approaches (genomics, transcriptomics, proteomics, and metabolomics), allows for the identification, characterisation, and quantification of all biological molecules that are involved in the structure, function, and dynamics of a cell, tissue or organism. A practical advantage of these approaches is that the use of human samples, such as tears and saliva, circumvent the use of other tissues that can only be obtained through invasive procedures [82,83].

Today, proteomics studies benefit from new features in mass spectrometry and gene data banks. Similarly, advances in peptide labelling methods allow for the identification and quantification of changes in protein expression in the entire proteome. Such astounding wealth of information can now be handled through ‘integromics’ [72], which is becoming a subfield in biostatistics and bioinformatics. Ultimately, such a comprehensive approach is promising, as it may identify novel therapeutic targets. For instance, with the use of mass spectrometry applied to proteomics, new biomarkers linked to renal damage have been identified for the diagnosis and prognosis of diabetic kidney disease. For instance, Bringans et al. [84] were able to develop a specific test that could predict the risk of developing nephropathy in type 2 diabetes, which in turn, opened up the possibility to update current standards of preventive care. Given its predictive value, it is of great scientific interest to take advantage of these novel molecular approaches to further our understanding of congenital urogenital conditions.

## Summary

The present systematic review showed that molecular biology approaches had been favoured over the years in search of hypospadias’ aetiology, even though close to half of these studies did not report hypospadias’ severity; sequencing and genotyping have been the preferred methods for research, and SNPs have been the most commonly reported findings.

## Key points

- From molecular biology approaches, a modest but growing number of studies have taken into account the severity of the condition in search of hypospadias’ aetiology.
- SNPs are the most frequently reported finding associated with hypospadias.
- Most genes associated with hypospadias fall into four gene-pathway categories related to androgens, oestrogens, growth factors, or transcription factors.
- In the future, additional information provided by molecular genetics screening prior to the urethroplasty procedure may nurture open discussions with parents about expected outcomes.
